# The Potential of Cylindromatosis (CYLD) as a Therapeutic Target in Oxidative Stress-Associated Pathologies: A Comprehensive Evaluation

**DOI:** 10.3390/ijms24098368

**Published:** 2023-05-06

**Authors:** Zhenzhou Huang, Yanjie Tan

**Affiliations:** Center for Cell Structure and Function, Shandong Provincial Key Laboratory of Animal Resistance Biology, Collaborative Innovation Center of Cell Biology in Universities of Shandong, College of Life Sciences, Shandong Normal University, Jinan 250358, China; huangzhenzhou97@163.com

**Keywords:** oxidative stress, CYLD, disease, deubiquitination

## Abstract

Oxidative stress (OS) arises as a consequence of an imbalance between the formation of reactive oxygen species (ROS) and the capacity of antioxidant defense mechanisms to neutralize them. Excessive ROS production can lead to the damage of critical biomolecules, such as lipids, proteins, and DNA, ultimately contributing to the onset and progression of a multitude of diseases, including atherosclerosis, chronic obstructive pulmonary disease, Alzheimer’s disease, and cancer. Cylindromatosis (CYLD), initially identified as a gene linked to familial cylindromatosis, has a well-established and increasingly well-characterized function in tumor inhibition and anti-inflammatory processes. Nevertheless, burgeoning evidence suggests that CYLD, as a conserved deubiquitination enzyme, also plays a pivotal role in various key signaling pathways and is implicated in the pathogenesis of numerous diseases driven by oxidative stress. In this review, we systematically examine the current research on the function and pathogenesis of CYLD in diseases instigated by oxidative stress. Therapeutic interventions targeting CYLD may hold significant promise for the treatment and management of oxidative stress-induced human diseases.

## 1. CYLD: An Overview

### 1.1. CYLD Structure and Function

Cylindromatosis (CYLD), belonging to the ubiquitin-specific protease (USP) family, is a deubiquitinase that selectively removes K63-linked ubiquitin chains and exhibits widespread distribution in vivo. Initially identified in familial cylindromatosis (FC), a skin appendage tumor typically manifesting on the scalp, CYLD is implicated in a genetic syndrome thought to originate from hair follicle stem cells [1]. Biggs et al. discovered the association between cylindromatosis and CYLD gene deletion on chromosome 16, subsequently determining the gene’s location on the long arm of chromosome 16 (16q12-13) in 1995 [2]. Multiple familial trichoepitheliomas (MFT) and Brooke–Spiegler syndrome (BSS) also exhibit loss of heterozygosity of CYLD [3]. FC, MFT, and BSS represent overlapping phenotypes resulting from CYLD deletion [3], underscoring the significance of CYLD as a crucial tumor suppressor.

CYLD, composed of 20 exons, encodes a protein containing 956 amino acids [4]. Its N-terminal region houses three CAP-Gly domains, which interact with targets such as NEMO in the NF-kB pathway [4]. These domains, originally identified in connections between endocytic vesicles and microtubules, comprise approximately 70 hydrophobic amino acid residues [5]. Functionally, CAP-Gly domains are present in several microtubule-binding proteins, including cytoplasmic adaptor protein CLIP-170 and dynactin1, and are postulated to facilitate the attachment of proteins like microtubule-binding proteins to microtubules [6]. Additionally, a ubiquitin-specific protease (USP) catalytic domain is located at the C-terminal region. This USP domain, capable of specifically removing K63-linked ubiquitin chains, also contains a B-box domain that mediates CYLD dimerization [7]. A small zinc finger binding module, akin to the E3 ligase B box and RING finger structure, is embedded within this domain, playing a role in CYLD subcellular localization [8].

### 1.2. The Cap-Gly Domains of CYLD and Microtubule-Related Cellular Processes

Research has revealed that the N-terminus of CYLD harbors three Cap-Gly domains, which are evolutionarily conserved motifs consisting of approximately 70 amino acids and an abundance of glycine residues [9]. A key function of the Cap-Gly domains is that two domains near the 3′ end can bind to the C-terminal EEY/F-COO(-) motifs of α-tubulin and certain microtubule-associated proteins, with the first Cap-Gly domain found to be necessary for this activity [10,11]. These domains regulate microtubule–tubulin interactions and suppress the deacetylation of tubulin by downregulating histone deacetylase-6 (HDAC6) or inhibiting its activity via trichostatin A (TSA), thereby influencing microtubule-related cell migration [11], cell cycle [12], and ciliogenesis [13]. The third CAP-Gly domain of CYLD specifically interacts with one of the two proline-rich sequences of NEMO/IKKγ in the NF-κB signaling pathway, a pathway that regulates gene expression involved in various biological processes such as development, inflammation, and tumorigenesis. CYLD-mediated NEMO deubiquitination impedes its phosphorylation of IκB, consequently inhibiting NF-κB signaling [14,15].

### 1.3. The USP Catalytic Structural Domain of CYLD and Deubiquitination Function

Ubiquitination has emerged as a crucial post-translational modification in diverse cellular processes, regulating protein degradation, autophagy, intracellular protein transport, DNA damage response, protein activation, and protein–protein interactions. Given that the deregulation of these processes can result in pathological conditions such as inflammatory diseases, neurodegeneration, or cancer, stringent regulation of the ubiquitin system is paramount [16]. CYLD proteins possess a USP catalytic structural domain at their C-terminus, which specifically removes Lys63 and Met-1-linked polyubiquitin chains [16] and disrupts protein interactions, leading to protein degradation by the protease system. This encompasses TNF receptor-associated factor 2 (TRAF2) and nuclear factor (NF)-κB essential modulator (NEMO), which are necessary for the canonical activation of NF-κB, and Bcl-37, which is required for noncanonical activation of NF-κB [7]. A B-box structure is embedded within the USP structural domain, and the deletion of the B-box does not significantly affect the deubiquitinase activity of CYLD. However, it disrupts CYLD intermolecular interactions, rendering the CYLD molecule incapable of localizing in the cytoplasm and binding to the ubiquitin chain complex [17].

## 2. CYLD and Oxidative Stress

### 2.1. Oxidative Stress and Associated Pathologies

Reactive oxygen species (ROS) are capable of inflicting damage upon lipids, nucleic acids, and proteins, subsequently altering their functionality [18]. Oxidative stress arises when an imbalance transpires between ROS production and the antioxidant defense mechanism [19]. A multitude of diseases, including atherosclerosis [20], chronic obstructive pulmonary disease (COPD) [21], Alzheimer’s disease [22], and cancer [23], have been associated with oxidative stress. For instance, cardiovascular disease, the foremost cause of death globally, is influenced by oxidative stress [24]. Elevated ROS levels lead to diminished nitric oxide availability and vasoconstriction, thereby promoting arterial hypertension [24]. Additionally, ROS negatively impacts myocardial calcium handling, instigates arrhythmias, and exacerbates cardiac remodeling by inducing hypertrophic signaling and apoptosis [25]. ROSs have also been implicated in the formation of atherosclerotic plaques.

Oxidative stress may function as an initiator in oocyte aging and reproductive pathologies, resulting in abnormal follicular atresia, aberrant meiosis, reduced fertilization rates, delayed embryonic development, and reproductive disorders such as polycystic ovary syndrome and ovarian endometriosis cysts [26]. Traumatic brain injury (TBI), a leading cause of mortality and morbidity worldwide, induces glutamate elevation at the synapse following a severe TBI event. Excess glutamate subsequently activates corresponding NMDA and AMPA receptors, promoting excessive calcium influx into neuronal cells. This cascade generates oxidative stress, culminating in mitochondrial dysfunction, lipid peroxidation, and oxidation of proteins and DNA, ultimately resulting in neuronal cell death [27].

ROS are implicated in various oncogenic processes, including initiation, promotion, activation, and inactivation of proto-oncogenes, as well as the stability and function of tumor suppressor genes [28]. Numerous studies have demonstrated that oxidative stress influences several signaling pathways linked to cell proliferation. Key signaling proteins, such as nuclear factor erythroid 2-related factor 2, RAS/RAF, mitogen-activated protein kinases ERK1/2 and MEK, phosphatidylinositol 3-kinase, phospholipase C, and protein kinase C, are affected by oxidative stress [29,30]. Moreover, ROSs modify the expression of p53 repressor genes, a crucial factor in apoptosis [31]. Consequently, oxidative stress induces alterations in gene expression, cell proliferation, and apoptosis, playing a significant role in tumorigenesis and progression. Table 1 enumerates other diseases induced by oxidative stress.

### 2.2. Function of CYLD in Oxidative Stress-Related Diseases

#### 2.2.1. Function of CYLD in Oxidative Stress-Induced Obesity-Related Nephropathy

With a rapidly increasing global prevalence of obesity, obesity has become a serious public health problem. In addition to predisposing to cardiovascular disease and diabetes [39,40], a growing number of reports suggest that obesity is also an important risk factor for kidney damage, namely obesity-related nephropathy (ORN) [40,41], which has become one of the major causes of end-stage renal disease.

**Figure 1:**
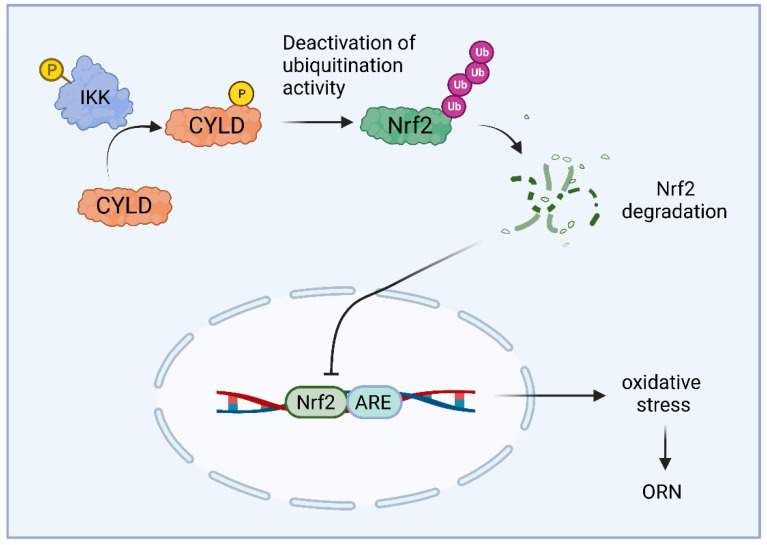
Schematic illustration of the mechanism through which IKK and CYLD phosphorylation mediates the activation of the Nrf2/ARE pathway, subsequently inducing oxidative stress in human kidney cells. IKK activates CYLD phosphorylation, which in turn inactivates CYLD’s deubiquitination activity. This promotes the ubiquitination of Nrf2, resulting in Nrf2 protein degradation and inhibition of the Nrf2/ARE signaling pathway. Consequently, oxidative stress is exacerbated in ORN-associated kidney injury.

Many studies have shown that oxidative stress is a characteristic of obesity [42] and is one of the main causes of kidney damage in ORN [43,44]. The imbalance between increased reactive oxygen species (ROSs) and/or decreased antioxidant activity promotes oxidative stress damage to tissues or cells [45,46]. ROS production induces glomerular and tubular damage, suggesting that ROSs play an important role in mediating renal injury, which may ultimately lead to the development of end-stage renal disease [47,48,49]. Therefore, reducing ROS production to ameliorate oxidative stress injury may be a new therapeutic target for ORN. IκB kinase (IKK) induces phosphorylation of CYLD. Phosphorylation serves as a mechanism to temporarily inactivate the deubiquitination activity of CYLD, thereby promoting the ubiquitination of its downstream molecules [50,51]. In one study, researchers found that oxidative stress damage was observed in the kidney tissue of ORN model mice and that IKK induced phosphorylation of CYLD, which in turn inactivated its deubiquitination activity [52]. Thus, phosphorylated CYLD instead promoted ubiquitination of Nrf2, which ultimately led to oxidative stress injury in ORN (Figure 1). These findings suggest that IKK promotes obesity-induced kidney injury via CYLD phosphorylation and that IKK inhibitors can alleviate lipid deposition and oxidative stress injury in ORN. Furthermore, IKK/CYLD/Nrf2 axis may provide a viable target for the treatment of ORN-induced kidney injury.

#### 2.2.2. Role of CYLD in Malignant Transformation of Tumors Resulting from Oxidative Stress-Induced DNA Damage

A vast majority of human cancers present persistent DNA damage and genomic instability as pathological features [53]. The accumulation of reactive oxygen species (ROS)-induced DNA damage, leading to genomic instability, is a crucial factor in the malignant transformation of tumors [53]. Several characteristic alterations transpire during cellular transformation, including autonomous proliferation, apoptosis evasion, invasion of surrounding tissues, and tumor metastasis [54]. These properties are concomitant with the aberrant activation of nuclear factor-kappa B (NF-kB) to bolster cancer cell survival and proliferation [55].

Oxidative stress-induced DNA damage may stimulate DNAPKsome assembly and Mps1 activation, which phosphorylates c-Abl at threonine 735 (T735) and promotes its cytoplasmic translocation [56]. Persistent cytoplasmic localization of c-Abl is associated with tumor cell transformation [57]. In addition, c-Abl phosphorylates OTULIN at tyrosine [58], disrupting its binding to LUBAC. The liberated LUBAC interacts with SPATA2 and is recruited to TNF-R1sc, promoting SPATA2-CYLD interactions [54]. These interactions are essential for oxidative stress to activate IKKβ, which in turn stimulates NF-κB transcriptional activity. IKKβ also induces phosphorylation of CYLD at serine 568, subsequently activating CYLD’s deubiquitination function to terminate NF-κB signaling [4].

**Figure 2:**
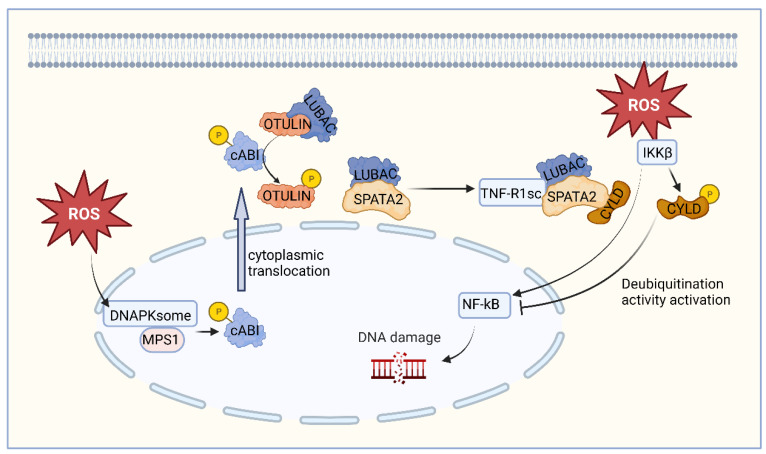
Schematic representation of the mechanism of CYLD function in oxidative stress-induced DNA damage. Oxidative stress-induced DNA damage may stimulate the assembly of DNAPKsome and the activation of Mps1, which phosphorylates c-Abl and promotes its cytoplasmic translocation. Also, c-Abl phosphorylates OTULIN, disrupting its binding to LUBAC. The released LUBAC interacts with SPATA2 and participates in the TNF-R1-mediated signaling pathway, promoting the interaction between CYLD and LUBAC. CYLD-LUBAC binding induces DNA damage by interacting with regulatory proteins and stimulating NF-kB activation. Additionally, oxidative stress activates IKKβ, which induces CYLD phosphorylation, in turn activating CYLD’s deubiquitination function and terminating NF-κB signaling.

Contrary to the prevailing notion of CYLD as a strict tumor suppressor, it initiates and terminates NF-κB activity by alternating between oncoprotein and tumor suppressor roles, respectively. Should IKKβ fail to induce the DUB activity of serine 568 phosphorylation, CYLD will persistently exhibit oncogenic activity [54]. The ensuing dysregulation of NF-κB activity and other associated pathological changes would disrupt intracellular homeostasis, thereby favoring tumor transformation (Figure 2).

#### 2.2.3. Role of CYLD in Ischemia-Reperfusion-Induced Liver Inflammation

**Figure 3:**
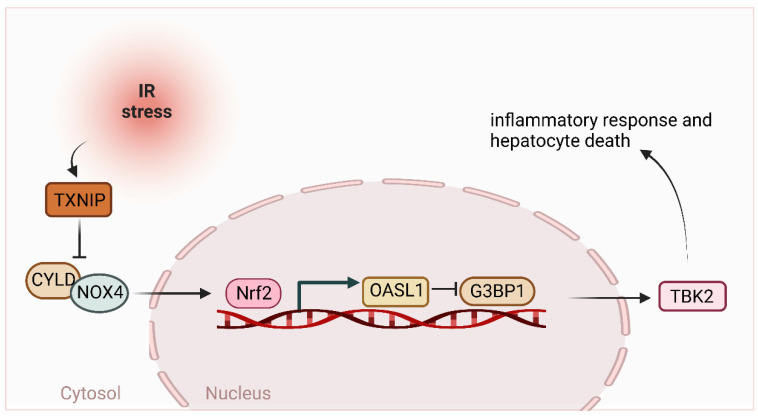
Schematic diagram of the mechanism of the role of CYLD in ischemia-reperfusion-induced liver inflammation. IR activates TXNIP to inhibit CYLD-NOX4 interaction, which activates NRF2 and its target gene OASL1 to suppress G3BP1 and subsequent TBK2-driven inflammatory responses and hepatocyte death.

Ischemia and reperfusion (IR)-induced liver inflammation and injury are important causes of liver dysfunction and failure after liver transplantation, resection, and hemorrhagic shock [59]. The IR-induced liver injury involves oxidative stress and endoplasmic reticulum (ER) stress-mediated inflammatory responses. Hepatic macrophages (Kupffer cells) are a key component of the liver’s innate immune system and are the first line of defense in detecting invading pathogens in the liver [60]. Activated macrophages produce reactive oxygen species (ROS) and initiate activation of the tophane-like receptor 4 (TLR4) or NLRP3 inflammasome, leading to liver inflammation and injury [59,61,62]. It has been shown that the knockdown of bone marrow-specific TXNIP ameliorates IR-induced liver injury and reduces macrophage/neutrophil accumulation and pro-inflammatory mediators in IR-stressed livers [63]. Macrophage TXNIP deficiency activates the NRF2-OASL1 pathway and regulates TBK1 function in IR-induced hepatitis injury. This study found that macrophage TXNIP deficiency promoted CYLD-NADPH oxidase 4 (NOX4) interactions and enhanced nuclear factor-like 2 (NRF2) and its target gene 2′,5′ oligoadenylate synthase-like 1 (OASL1) activity, leading to IR stress-induced liver injury inhibiting Ras-GTPase-activating protein binding protein 1 (G3BP1) and TBK1-driven inflammatory response and hepatocyte death [63]. Thus, the molecular regulatory mechanisms of the macrophage TXNIP-mediated CYLD-NRF2-OASL1 pathway in the IR-stressed liver may provide potential therapeutic targets for stress-induced liver inflammation and injury (Figure 3).

#### 2.2.4. CYLD Enhances Oxidative Stress in the Heart

In response to pathological stresses, such as pressure overload and other myocardial injuries, the heart initially activates an adaptive physiological hypertrophic response. However, a sustained cardiac hypertrophic response can lead to pathological cardiac hypertrophy, fibrosis, and cell death, ultimately resulting in heart failure and death [64]. Oxidative stress is a state of excessive intracellular ROS levels, which can cause DNA damage, lipid peroxidation, and protein aggregation, leading to pathological cardiomyocyte hypertrophy and death in the heart [65].

Recently, CYLD expression was found to be significantly upregulated in cardiomyocytes from hypertrophied and failing human and mouse hearts. CYLD knockout improved survival in mice and attenuated myocardial hypertrophy, fibrosis, apoptosis, oxidative stress, and dysfunction due to sustained pressure overload caused by transverse aortic constriction [52]. The most significantly altered genes revealed by sequencing and gene array analysis were those involved in free radical scavenging pathways and cardiovascular disease, including fos, jun, myc, and nuclear factor-erythroid-2-related factor 2 (Nrf2) in the heart. Nrf2 is an essential negative regulator of oxidative stress in cardiomyocytes, thereby inhibiting cardiac rational remodeling and dysfunction in different pathological settings [66,67,68,69].

**Figure 4:**
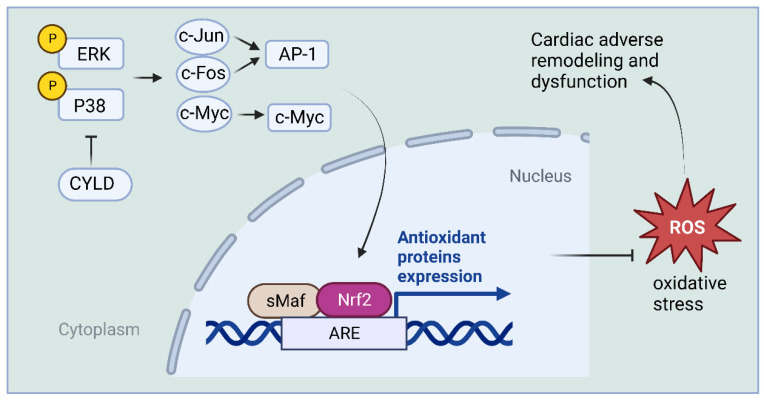
Schematic representation of the mechanism by which CYLD enhances oxidative stress in the heart. CYLD inhibits the antioxidant capacity of Nrf2 by blocking ERK- and p38-/AP-1, as well as c-Myc pathways, consequently enhancing oxidative stress in the heart.

This study found that CYLD knockdown enhanced the expression of mitogen-activated protein kinase (MAPK) ERK- and p38-mediated c-jun, c-fos, and c-myc that control Nrf2 expression in cardiomyocytes. The inhibition of cardiomyocyte reactive oxygen species (ROS) formation, death, and hypertrophy by CYLD deficiency was blocked by Nrf2 knockdown [52]. Thus, CYLD mediates cardiac maladaptive remodeling and dysfunction, likely by enhancing myocardial oxidative stress in response to stress overload. CYLD blocks ERK-, p38-/AP-1, and c-Myc pathways and inhibits the antioxidant capacity of Nrf2, thereby enhancing oxidative stress in the heart (Figure 4).

#### 2.2.5. Role of CYLD in Oxidative Stress-Induced Retinal Pigment Epithelial Cell Damage and Dysfunction

Age-related macular degeneration (AMD) is a chronic and progressive degenerative disease of the retina that ultimately results in blindness [70]. Retinal pigment epithelial (RPE) cell damage and dysfunction induced by oxidative stress are significant pathogenic factors in AMD [71]. During oxidative stress, CYLD-AS1 expression is upregulated in RPE cells. Depletion of CYLD-AS1 promotes cell proliferation and mitochondrial function, protecting RPE cells from hydrogen peroxide (H_2_O_2_)-induced damage. CYLD-AS1 also modulates the expression of members of the NRF2 and inflammation-related NF-κB signaling pathways, which are associated with oxidative stress. These two signaling pathways are mediated by the CYLD-AS1 interactor, miR-134-5p. CYLD-AS1 influences the oxidative stress-related inflammatory function of RPE cells by sponging miR-134-5p-mediated NRF2/NF-κB signaling pathway activity [72]. Consequently, targeting CYLD-AS1 may represent an effective strategy for the treatment of AMD-related diseases.

## 3. Other Biological Functions of CYLD

### 3.1. Function of CYLD in Ciliary Diseases

Cilia are highly specialized cellular structures that protrude from the surface of the cell membrane and are found in a wide range of organisms, from unicellular eukaryotes to vertebrates. They sense extracellular signaling molecules and regulate various cellular activities [73]. Defects in the structure and function of primary cilia cause a range of diseases, including polycystic kidney disease, microcephaly, retinal degeneration, obesity, liver dysfunction, polydactylism, neurological disorders, and malignancy, collectively known as ciliopathy [74]. CYLD plays a crucial role in matrix anchoring and assembly of primary and motor cilia in several organs. Ciliopathy phenotypes, including male sterility, impaired lung maturation, and osteoporosis, have been observed in Cyld-deficient mice. CYLD is required for ciliogenesis, and Cyld-knockout mice exhibit defects in multiple organs, including skin, kidneys, trachea, and testis [13]. Transmission electron microscopy has shown that anchoring of the matrix and proper assembly of the matrix and axon require CYLD. CYLD deubiquitinates the 70 kDa central protein (Cep70), thereby increasing the Cep70 localization of the matrix, facilitating matrix organization and anchoring to the plasma membrane. Additionally, CYLD-mediated inactivation of HDAC6 enhances microtubule protein acetylation, stabilizing axonal microtubules and promoting cilia formation [13]. Some studies have also shown that CYLD locates in the centrosome and basal body through its interaction with the centrosome protein CAP350. When the interaction between the two proteins is eliminated, matrix migration docking is damaged, resulting in cilia loss [75]. Furthermore, CENPV, a component of mitotic chromosomes associated with cytoplasmic microtubules, interacts with CYLD through the CAP-Gly structural domain and is deubiquitinated by CYLD to promote cilia formation [76]. Many studies have shown that CYLD gene deletion mice exhibit various cilia-related diseases [77,78,79], and the treatment of cilia diseases by CYLD warrants further investigation.

### 3.2. Function of CYLD in Neuronal Development

Abnormal fear memory is a hallmark of many neuropsychiatric disorders [80]. Proper neuronal activation and excitability in the basolateral amygdala (BLA) are necessary for the formation of fear memories [81]. It has been shown that Cyld knockdown impairs amygdala-dependent tone-cued fear memory [82]. Cyld is expressed in several brain regions, including the amygdala. Cyld deficiency leads to abnormal neuronal excitation, with reduced frequency of spontaneous excitatory postsynaptic currents and amplitude of microexcitatory presynaptic currents in BLA principal neurons. It has been demonstrated that CYLD deficiency disrupts neuronal activity and synaptic transmission in the BLA of mice, potentially leading to impaired fear memory. Auditory neuropathy is an important cause of hearing loss [82]. It has been shown that CYLD-KO mice have mild hearing impairment. CYLD is widely expressed and localized in cochlear tissue in vitro and in various neuronal cell models. Knockdown of CYLD reduces the length and proportion of neurite outgrowth in neuronal cells. The abnormal hearing in Cyld KO mice may be caused by a reduction in the length and number of neurite outgrowths in auditory neurons in the cochlea. This suggests that CYLD is a key protein affecting hearing. Proteomic analysis of rodent brain samples also shows that CYLD is partially enriched in purified postsynaptic densities. CYLD regulates dendritic growth and postsynaptic differentiation in mouse hippocampal neurons [82].

### 3.3. Function of CYLD in Vascular Disease

Arteries transport blood from the heart to other organs, and their walls are composed of three layers from the lumen to the exterior: the inner membrane, the middle membrane, and the outer mold. The endothelium consists mainly of endothelial cells, a few fibroblasts, smooth muscle cells, and sparse elastic fibers; the mesothelium consists mainly of smooth muscle cells interspersed with fibroblasts; and the outer mold is mainly collagen fibers and fibroblasts, with an elastic layer separating the mesothelium from the endothelium and outer mold [83,84]. Studies have shown that CYLD affects vascular disease in several ways.

Rac1 is a Rho family of GTPases that regulates actin cytoskeleton and adhesion rearrangement and plays a key role in cell polarization and migration [85]. CYLD deubiquitinates Rac1 and promotes Rac1 activation for endothelial cell migration and angiogenesis [86]. CYLD expression in endothelial cells (ECs) and macrophages decreases with age, exacerbating monocyte adhesion to endothelial cells and foam cell formation, triggering the development of age-related atherosclerosis. Therefore, the CYLD gene in the vasculature may be a new therapeutic target in early intervention to prevent age-related atherosclerosis formation [87].

It has been shown that CYLD mediates a pro-inflammatory phenotype of vascular smooth muscle cells (VSMC) through MAPK activation, characterized by loss of contractility, apoptosis, production of extracellular matrix and cytokines, and foam cell-like transformation, which may contribute to the development of coronary artery lesions [88]. Pulmonary arterial hypertension (PAH) is a common complication of congenital heart disease (CHD), and CYLD mediates human pulmonary artery smooth muscle cell (HPASMC) dysfunction, which regulates HPASMC phenotypic transformation, proliferation, and migration through modulation of p38 and ERK activation [89]. CYLD is a potential new therapeutic target for the prevention of PAH and pulmonary vascular remodeling in CHD-PAH.

Transdifferentiation of extravascular fibroblasts (AFs) to myofibroblasts plays a key role in atherosclerosis, postoperative restenosis, and vascular remodeling in aortic aneurysms [90]. Nicotinamide adenine dinucleotide phosphate oxidase 4 (Nox4), a member of the NADPH oxidase family, is a major source of ROS in the vascular wall. AFs have been shown to produce large amounts of NADPH oxidase-derived ROS in response to vascular injury [6]. CYLD promotes the transdifferentiation of AFs by directly binding to Nox4 through the USP structural domain, which plays a role in vascular remodeling, and CYLD can be used as a new target for vascular anti-inflammatory therapy for diseases such as abdominal aortic aneurysms [91]. In addition, CYLD-mediated deubiquitination of IκB kinase C (IKKγ), IκBα, or TNF receptor-associated factor 2 (TRAF2), leading to inhibition of NF-κB activity, is thought to resolve the vascular inflammatory response and thus inhibit vascular injury [92].

### 3.4. The Role of CYLD in Nephropathy

Diabetic nephropathy (DN) is a primary cause of chronic kidney disease (CKD). Podocytes, the end-differentiated epithelial cells of the glomerulus, are vital for maintaining an intact glomerular filtration barrier (GFB). Damaged podocytes are a crucial factor in the development of proteinuria and DN [93]. A contributing factor to podocyte damage is the destruction of actin and intermediate filaments resulting from mitochondrial damage [94]. RING-finger protein 166 (RNF166) is a member of the E3 ubiquitin ligase family. It has been demonstrated that RNF166 can directly interact with CYLD to regulate CYLD degradation. Overexpression of CYLD following RNF166 gene knockdown eliminated mitochondrial dysfunction and apoptosis of podocytes under high glucose stimulation due to RNF166 knockdown. Consequently, maintaining the protein level of CYLD in podocytes by inhibiting RNF166 expression or promoting CYLD expression might be a promising therapeutic strategy for DN treatment [78].

### 3.5. CYLD and Cancer

As a tumor suppressor gene, the expression level of CYLD plays a crucial regulatory role in tumorigenesis (Table 1). Most solid tumors exhibit infiltration of immune and inflammatory cells. Inflammation is a hallmark of cancer and plays a key role in cell transformation, invasion, metastasis, and treatment resistance [95,96]. Certain factors in CYLD regulation can also influence tumorigenesis. MicroRNAs (miRNAs) are a class of small non-coding RNAs consisting of 17–25 nucleotides. The miRNAs target the 3′-untranslated region (UTR) of mRNA or other non-coding RNAs and interact with AGO proteins to form RNA-induced silencing complexes (RISCs) that inhibit the expression or degrade target genes [97]. In non-small cell lung cancer (NSCLC), microRNA-135b (miR-135b) directly targets the 3′-untranslated region (UTR) of the deubiquitinase CYLD, thereby regulating the ubiquitination and activation of NF-κB signaling [98], promoting lung cancer cell proliferation, migration, invasion, anti-apoptosis, and angiogenesis. MicroRNA-587 (miR-587) can exacerbate NSCLC by downregulating CYLD and promoting the proliferation and migration ability of NSCLC [99]. Additionally, long non-coding RNAs (lncRNAs) can regulate malignant tumor initiation, development, and metastasis [100]. The LncRNA-LINC01260 gene can suppress NSCLC tumorigenesis through competitive endogenous RNA and ultimately inhibit NF-κB pathway activation by regulating CYLD expression, providing a potential target for NSCLC treatment [101]. Defective CYLD expression or function may profoundly impact the growth and survival of various cancer cell types, with some cancer types associated with CYLD function listed in Table 2. Therefore, CYLD may be considered a new target for cancer therapy.

## 4. CYLD Is a Potential Therapeutic Target for Disease

### 4.1. CYLD Is a Potential Therapeutic Target for Autism Spectrum Disorder and Parkinson’s Disease

Current treatment options for autism spectrum disorder (ASD) and Parkinson’s disease (PD) remain limited in their efficacy [115]. Clinical trials aimed at improving the lives of affected individuals have delivered disappointing outcomes. For instance, the Phase 3 clinical trial of arbaclofen, a GABA-B receptor agonist, failed to show significant improvements in social function in patients with ASD [21]. Likewise, the trial of the phosphodiesterase-10A inhibitor, PF-02545920, did not show promising results in improving motor symptoms in PD patients [116]. Recent studies on the CYLD have provided intriguing insights into its role in the pathophysiology of ASD and PD. While the potential of targeting CYLD as a therapeutic option is promising, several challenges must be addressed. For instance, the development of CYLD inhibitors or modulators of the pathways it is involved in may face obstacles related to off-target effects, toxicity concerns, and the complexity of the underlying molecular networks.

In the context of ASD, CYLD has been implicated in the regulation of mechanistic target of rapamycin (mTOR) signaling, synaptic α-amino-3-hydroxy-5-methyl-4-isoxazolepropionic acid (AMPA) receptor subunits, and autophagy in the hippocampus. However, the mTOR signaling pathway is complex and has a plethora of downstream targets that could be affected by the inhibition of CYLD, raising concerns about the potential off-target effects and the risk of dysregulating other physiological processes [117,118]. Similarly, the development of CYLD inhibitors for PD treatment may encounter challenges related to the PINK1/parkin pathway’s multifaceted nature, which regulates mitochondrial quality control and has implications for cellular homeostasis beyond neurodegeneration. Inhibition of CYLD may influence other proteins within this pathway, potentially leading to unforeseen consequences or toxicity concerns [119].

Although no specific CYLD inhibitors have been developed to date, the search for molecules capable of modulating CYLD activity is ongoing. Some potential CYLD inhibitors, such as PR-619, have shown promising results in vitro by reducing CYLD activity and rescuing mitochondrial dysfunction in cellular models of PD [115]. Additionally, the development of small-molecule modulators targeting CYLD-related pathways, such as mTOR inhibitors like rapamycin and its analogs, has demonstrated therapeutic potential in preclinical models of ASD [120]. These findings suggest that CYLD inhibition, either directly or indirectly, could pave the way for innovative treatment options for ASD and PD.

The efficacy of CYLD-targeted therapies can be evaluated using a combination of in vitro and in vivo models, as well as clinical trials. For example, cellular models of ASD and PD can be employed to assess the impact of CYLD inhibition on neuronal connectivity, synaptic function, and mitochondrial quality control. Furthermore, animal models, such as transgenic mice, can provide valuable insights into the therapeutic potential of CYLD inhibitors in alleviating behavioral and motor deficits. Ultimately, well-designed clinical trials are necessary to establish the safety, tolerability, and efficacy of CYLD-targeted therapies in patients with ASD and PD.

### 4.2. Drugs Targeting Pathways Implicated in CYLD Dysfunction

The development of small molecule drugs targeting signaling pathways implicated in CYLD dysfunction has gained significant interest, as these drugs hold promise for modulating dysregulated cellular processes associated with CYLD mutations and related diseases [121,122]. CYLD, a deubiquitinating enzyme, negatively regulates pathways such as the NF-κB, Wnt/β-catenin, and JNK pathways through the removal of K63-linked ubiquitin chains from target proteins [8,123]. Small molecule inhibitors targeting these pathways function by modulating specific components of the pathways, potentially ameliorating the effects of CYLD dysfunction. For the NF-κB pathway, BMS-345541 selectively inhibits the ATP-binding site of IKK, preventing IκB phosphorylation and degradation and ultimately inhibiting NF-κB activation [124]. Bortezomib, a proteasome inhibitor, prevents IκB degradation, thereby indirectly inhibiting NF-κB activation [125,126]. In the Wnt/β-catenin pathway, LGK974 and ETC-159 function as porcupine inhibitors, blocking the secretion of Wnt proteins essential for pathway activation [127,128]. This inhibition disrupts the activation of downstream signaling components, such as Dishevelled (Dvl) and β-catenin [129]. Tankyrase inhibitors, on the other hand, stabilize axin, a negative regulator of the Wnt/β-catenin pathway, leading to decreased β-catenin levels and pathway inhibition [130]. Regarding the JNK pathway, SP600125 inhibits JNK activation by targeting the ATP-binding site of the kinase, preventing the activation of downstream targets involved in cell proliferation and survival [131].

These small molecule inhibitors have shown potential in preclinical studies and early-phase clinical trials, with some, like bortezomib, already approved for specific clinical indications [132,133]. However, their safety profiles and potential side effects, which may include gastrointestinal symptoms, fatigue, hematological toxicities, and hepatotoxicity, need to be carefully evaluated. Additionally, the context-dependent effectiveness of these drugs warrants further investigation, as factors such as disease stage, genetic background, and the presence of other molecular alterations can influence their therapeutic outcomes [134]. Continued research is essential to optimize the safety and efficacy profiles of these small molecule drugs, enabling their use as potential therapeutic options for patients with CYLD-related diseases and broadening our understanding of the intricate interplay between CYLD and its associated signaling pathways.

## 5. Conclusions

Oxidative stress (OS) is an imbalance between reactive oxygen species (ROS) formation and antioxidant defense mechanisms and affects the normal function of multiple tissues. Many age-related chronic diseases, such as diabetes and cardiovascular, renal, pulmonary and skeletal muscle diseases, are also directly associated with OS. Although many of the small molecules evaluated as antioxidants have shown therapeutic potential in preclinical studies, clinical trial results have been disappointing. Therefore, many studies are also trying to elucidate the potential mechanisms and role of OS in disease onset and progression and to find new therapeutic strategies to reduce OS. CYLD encodes a deubiquitinating enzyme that is a key regulator of various cellular processes, including immune response, inflammation, death and proliferation, and directly regulates several keys signaling cascades, such as NF-kB and MAPK pathways, involved in the development of multiple diseases, including cancer, poor infection control, pulmonary fibrosis, neurodevelopment and cardiovascular dysfunction. This review explores the functional and mechanistic studies of CYLD in oxidative stress-induced diseases, which provide a strong rationale for the design and testing of specific CYLD inhibitors that may have translational potential for the treatment of oxidative stress-related diseases. In conclusion, targeting CYLD and its associated pathways holds promise as a novel therapeutic strategy for oxidative stress-induced diseases. However, significant challenges remain, including the development of specific CYLD inhibitors, potential off-target effects, and toxicity concerns. Future research should focus on addressing these issues and elucidating the precise mechanisms by which CYLD modulation could ameliorate the symptoms of these complex disorders.

In addition to the loss of function in human disease tissues through gene deletion or mutation, CYLD expression can also be regulated at the RNA level through transcriptional regulation or at the protein level through post-translational modifications, if necessary. The identification of CYLD-mediated signaling pathways during disease progression will also provide a solid basis for diagnosis and facilitate the development of new tools for disease treatment. We expect that all of these approaches will help to advance antioxidant therapy and hope that this review will encourage and inform a sound approach to this worthwhile endeavor.

## Figures and Tables

**Table 1:** Oxidative Stress-Induced Diseases.

Disease	Mechanism	Reference
Alzheimer’s	promotes Aβ deposition, tau hyperphosphorylation, and the subsequent loss of synapses and neurons	[22]
chronic kidney disease	antioxidant depletions and increases ROS production	[32]
periodontitis	increases ROS production	[33]
male infertility	damages sperm DNA, RNA transcripts, and telomeres	[34]
osteoporosis	diminishes bone mineral density in osteoporosis	[35]
endometriosis	causes a general inflammatory response in the abdominal cavity	[36]
vitiligo	damages melanocytes by ROS	[37]
nonalcoholic fatty liver	increases ROS production	[38]

**Table 2:** The relationship between CYLD and cancer.

Function	Cancer Relevance	Reference
phosphorylation	lymphoma, breast cancer, B-cell lymphoma	[51,102,103]
deubiquitination	prostate cancer, nasopharyngeal carcinoma, cancer of the stomach, lung cancer	[98,104,105,106]
mutation	basal cell salivary gland tumor, skin cancer, squamous cell carcinoma of the head and neck	[107,108,109]
defect	multiple myeloma, melanoma	[110,111]
transcriptional inhibition	colon cancer, liver cancer	[112,113]
regulatory microtubule/tubulin	pancreatic cancer, leukemia	[6,114]

## Data Availability

Not applicable.
